# To Junior Dentists

**Published:** 1886-12

**Authors:** 


### TO JUNIOR DENTISTS. NO. VII.

### TAKING IMPRESSIONS OF THE MOUTH.

*My Dear Doctor:*

Your letter asking advice in the taking of impressions of the mouth is at hand, and I answer it thus publicly in the hope that others may derive some benefit from what I have to say. I confess that I do not know half as much about the subject as many, but if they keep their knowledge to themselves instead of imparting it through the journals, it only remains for one less qualified to give what information he can.

The first and most important step in the making of an artificial denture is to secure a perfect impression of the parts to which the plate is to be fitted, for it matters not how excellent may be the workmanship of the finished piece, it can never be of any practical utility unless adaptation be perfect. All the polishing and finishing which man is capable of giving a denture will not serve in place of accuracy of fit. In speaking of taking impressions, then, I am going back to the initial point and dealing with primal principles.

The securing of a proper counterpart or *fac-simile* of the arch of a mouth of uniform hardness throughout, with no teeth remaining, is a comparatively simple matter. But when there are teeth remaining in the jaw, some of them with bell-shaped crowns, the alveoli absorbed away for one-third the length of the roots and the teeth possibly inclined at an angle several degrees removed from the perpendicular, or where, in a jaw that has long been without teeth, the central portions of the arch are perfectly unyielding while the alveolar ridge is soft and flexible, the obtaining of a proper working model is a matter requiring a high degree of skill. In the former case, to obtain a correct impression of the tissue about the cervical portions of the teeth and to get a perfect model of the irregular teeth themselves, is a problem that few have quite solved. I know many skillful dentists who content themselves with an approximately correct model, which they carve to their idea of the true condition. I need not say that this cannot be exactly accomplished.

When there are soft and spongy portions of the arch in full cases, it is not sufficient to obtain a reproduction in plaster-of-paris or metal of the tissues in their normal state of rest. If there be a hard median ridge with a yielding border, the pressure of occlusion upon one side in mastication will cause the dislodgment of the plate upon the opposite side, and so there will be a continuous rocking of the denture upon this immovable central ridge, and it will be a failure. These two conditions are the ones which give to dentists the greatest annoyance, and it is to these complications that I shall devote my principal attention.

There are a number of different materials used for the securing of an impression of the mouth, the principal of which are plaster-of-paris, wax, gutta-percha, and the various forms of modeling compound. While each presents certain advantages in peculiar cases, I myself have long since abandoned all except the first, save that in a very few exceptional instances I sometimes employ the last. Wax would, without doubt, give the most perfect impression, were it possible to avoid the displacement of the thin portions in withdrawing it. Gutta-percha would be admirable only for its fatal tendency to "draw." Modeling compound, if left to harden in the mouth, makes a capital material, but it is impossible to remove it from around bell-shaped teeth without destroying the adaptation. Plaster-of-paris alone, when it has set, is free from these objections, but the difficulty in partial impressions is to withdraw it from the mouth, while in full cases, where there is a hard center with a yielding ridge, it is not possible with it alone to obtain a model that shall give a properly balanced denture. Twenty years of experience in taking impressions has enabled me to overcome some of these difficulties, and I will give you the results of those years of defeats and triumphs.

First, let me speak of the taking of partial impressions. It is absolutely essential that you possess a good stock of impression cups, or trays. For years I have bought every new form that has been offered, and every one has, at one time or another, found its appropriate place. I use, however, none but those made of britannia metal. An inflexible, unyielding cup has no place in my laboratory. But of the ordinary forms I have a large number, and I seldom take an impression without in some way changing the shape of the selected cup, either by cutting away a portion, or by bending it so that it will still better fit about the teeth. When, for the time, I am through with plate-shears and plyers, some common impression wax is warmed and made pliable in hot water, a sufficient quantity is placed in the cup and a wax impression is secured. This is the work of but a moment, and if this shows me that my cup is not properly fitted, it is again bent or cut away wherever the impression shows me that there exists the necessity.

If changes have been necessary, I place more wax in the cup and take another impression, and this is continued until I get the cup to my liking, when a final wax impression is taken, the wax being quite soft and the cup pressed firmly against the teeth as far as it will go. This is removed and, while it is yet pliable, the outer rim is bent back to place, if it be displaced, and somewhat beyond, the marks of the cup being my guide. This will allow a little space between the outer portion and the teeth, or the alveolar border, when it is replaced in the mouth.

I now take the impression to the laboratory, and with a hot knife I trim away all the surplus of wax, outside the teeth cutting down to the edge of the cup while the rim is left higher opposite the space to be occupied with artificial teeth. With a knife having a narrow blade I enlarge the sockets made by the natural teeth, until if replaced they would not be in contact with the wax, save upon the summit of their crowns. I also cut away the wax in the center of the arch to the depth of an eighth of an inch or more, and to a line about a quarter of an inch from the teeth and the posterior border of the wax impression. It is then, with the back of my wax knife, scarified all over the palatal portion, and longitudinally within the outer border and in the sockets made by the natural teeth. I hold the cup over a Bunsen burner or alcohol lamp until the metal is heated sufficiently to melt the wax a little over the entire surface of the cup, when it is held under the cold water faucet until cold and hard.

If this has been carefully done, I now have a cup that is perfectly adapted to my case, made of a material which will yield a little when I wish it to do so. Just enough of a rather thin batter of plaster-of-paris is now placed in it to nicely fill my manufactured cup, but which will allow no surplus to embarrass me, and this is carried quickly to the mouth. As soon as the plaster remaining in the mixing-cup will break with a clean fracture, and while it is yet soft enough to be a little waxy, I commence my manipulation for its removal, and just here is where the key-note of success is sounded. The operator must learn by experience at just what point to begin. If he commence too soon his plaster will crush, and his impression will be destroyed. If he delay a moment too long he will not, in difficult cases, be able to remove it without destroying it.

There will be instances of converging teeth from between which it will be impossible, without practical destruction of the impression, to remove the plaster-of-paris. In such very exceptional cases I place a piece of softened modeling compound in the space, which is allowed to remain until after the impression is taken. It is then removed and put in position before running the cast.

I commence removal by gently "working" the cup, at first up and down until it commences to yield, and then laterally until it is quite loose, when it is easily withdrawn. It is impossible to give you exact directions for this process. Your own good judgment and a little experience will guide you. Of course the plaster will not allow the cup to be withdrawn, in difficult cases, until it has been fractured, and my object is to break it away from undercuts and from bell-shaped teeth, crowding it back into the wax, which will yield for that purpose, until the whole can be removed. The outer rim will, in many cases, be quite broken away from the rest, and now are apparent the advantages of using the wax.

First. It gives me a cup that is exactly adapted to the case, and one which requires but a minimum amount of plaster.

Second. It affords me a yielding base for my plaster, and allows it to break about the teeth without comminution, at the same time retaining all the pieces in near apposition and preventing their loss.

Third. It affords me an unfailing guide in replacing any broken portions.

The scarification assists the adherence of the plaster to the wax and precludes their separation, besides assisting me in replacing broken pieces. The melting of the wax to the cup prevents the separation of the whole and enables me to use considerable force in removal, if it be necessary.

The essential point is to know just when to commence the removal. If it be done at the right moment, the whole is easily accomplished, and a perfect impression will be secured in cases that would seem to be impracticable. But a moment too soon or too late will end in a failure. Ten persons will, however, commence too late, for one who begins too quickly. Remember, your plaster must not be quite hard.

I need not say that, to obtain a perfect impression of the cervical borders of teeth, it is essential that they be clean and as dry as you can get them. Look to that before putting the cup in the mouth.

I have taken up so much space already that I can but allude to the second class of difficult cases—those in which, with a hard median portion, the sides of the vault, or the summit of the alveolar ridge, are spongy and soft. For these cases, in taking the wax impression, the material is used in a firmer state, so that a little pressure is necessary to force it to place. The edges only of the rim are bent back a little, and the surplus is trimmed away. From only such portions as covered the hard, unyielding parts of the mouth, the wax is removed to a depth dictated by the amount of difference in the yielding character of the tissues, when it is scarified, melted to the cup, and a rather stiff batter of plaster used, the cup being pressed firmly to place and held so until the plaster is firmly set. This will produce sufficient pressure upon the soft tissues to condense them all that is necessary.

The advantages of the wax in these cases are :

First. The ability to produce pressure upon the soft tissues.

Second. There being but little plaster used, the operation is much neater.

Third. The plaster is confined just where it is wanted.

Fourth. The folds of the buccal and lingual tissues, which are so likely to get beneath the cup when plaster only is used, are invariably pushed to one side.

Fifth. The stratum of plaster-of-paris is nearly uniform in thickness all over the mouth, and hence there is no unequal expansion in the setting, or crystallization.

